# Perioperative nursing coordination and complication management in Yttrium-90 radioactive microsphere therapy for hepatocellular carcinoma

**DOI:** 10.3389/fmed.2025.1694152

**Published:** 2025-12-04

**Authors:** Xian Li, Qiangwei Shang

**Affiliations:** Department of Hepatobiliary Surgery, Qilu Hospital of Shandong University, Jinan, Shandong, China

**Keywords:** Yttrium-90 microspheres, hepatocellular carcinoma, interventional radiology, perioperative nursing, complication management

## Abstract

To explore the perioperative nursing protocols and complication management strategies for hepatocellular carcinoma (HCC) patients undergoing Yttrium-90 (Y-90) radioactive microsphere therapy.

**Methods:**

A retrospective analysis was performed on clinical data from 15 HCC patients treated with Y-90 radioactive microspheres at Qilu Hospital of Shandong University between December 2024 and April 2025. A structured, multidisciplinary perioperative nursing protocol was implemented, consisting of: (1) Preoperative multidisciplinary evaluation including nutritional risk screening (NRS-2002), radiation dosimetry assessment, and vascular anatomy verification; (2) Intraoperative coordination with specific steps for patient positioning, vascular access establishment, radiation safety measures, and vital sign monitoring; (3) Postoperative dynamic monitoring of vital signs (every 2 h), puncture site condition, laboratory parameters (at 24 and 72 h), and symptom assessment using NRS for pain; (4) Tiered complication management system based on CTCAE v5.0 grading criteria. All 15 patients completed the treatment successfully. The postoperative complication rate was 73.3% (11/15), with manifestations including abdominal pain (five cases), fatigue (three cases), leukopenia (three cases), thrombocytopenia (two cases), and low-grade fever (one case), all complications resolved with appropriate symptomatic management. No severe adverse events such as radiation-induced liver injury or significant myelosuppression occurred. Standardized perioperative nursing workflows, comprehensive radiation protection protocols, and an early-warning system for complications are essential to enhancing the safety of Y-90 microsphere therapy.

## 1 Introduction

Hepatocellular carcinoma (HCC), as the main pathological type of primary liver cancer, accounts for 75%–85% of global liver cancer cases, with its incidence and mortality remaining high for many years. The latest statistics from the World Health Organization (WHO) show that in 2022, there were 870,000 new cases of liver cancer worldwide and 760,000 deaths, ranking third among the causes of cancer-related deaths (1). In China, there were 370,000 new cases of liver cancer (4th in incidence) and 320,000 deaths (2nd in mortality). Radical liver resection and liver transplantation remain the core methods for HCC patients to achieve potential cure, but clinical practice faces severe challenges: approximately 70% of patients lose the opportunity for surgery because they have progressed to intermediate or advanced stages (BCLC B/C) at initial diagnosis or have severe decompensated cirrhosis (2). Even after surgery, the 5-year recurrence rate remains as high as 60%–70%, urgently requiring the development of more effective comprehensive treatment strategies (3).

In recent years, with breakthroughs in targeted therapy, immune checkpoint inhibitors (such as PD-1/PD-L1 monoclonal antibodies), and local interventional techniques, the treatment landscape for HCC is undergoing profound changes. The “atezolizumab + bevacizumab” regimen based on the IMbrave150 study results has extended the median survival of advanced HCC to 19.2 months, becoming the new first-line treatment standard (4); while interventional treatments represented by transarterial chemoembolization (TACE) and hepatic artery infusion chemotherapy (HAIC) have further improved objective response rates by combining targeted/immunological drugs (such as TACE + lenvatinib + sintilimab) (5). Nevertheless, the heterogeneity, drug resistance, and treatment-related liver damage in intermediate and advanced HCC remain key bottlenecks restricting the improvement of efficacy. In this context, Yttrium-90 (Y-90) radioactive microsphere selective internal radiation therapy (SIRT), with its precise radiobiological effects and minimally invasive advantages, provides a new treatment option for patients with unresectable HCC, but its clinical application still faces the dual challenges of radiation protection and complication management. Complications were defined and graded according to the Common Terminology Criteria for Adverse Events (CTCAE v5.0). The tiered management approach included: Grade 1 (mild): symptomatic support and monitoring; Grade 2 (moderate): pharmacological intervention; Grade 3–4 (severe): multidisciplinary escalation and specialized care. The early-warning system was triggered by specific thresholds: NRS pain score ≥ 4, temperature > 38 °C, WBC < 3 × 10^9^/L, or ALT/AST > 3 × upper limit of normal.

Yttrium-90 radioactive microsphere selective internal radiation therapy (SIRT) is an important treatment for intermediate and advanced liver cancer and metastatic liver cancer. SIRT refers to radioactive microspheres injected into the hepatic artery, where they lodge in the blood vessels supplying the tumor and release beta rays to precisely kill the tumor. Y-90 resin microspheres were approved by the U.S. Food and Drug Administration (FDA) in 2002 for the treatment of colorectal liver metastases (CRLM), and Y-90 glass microspheres were officially approved by the FDA for HCC treatment in 2021. Yttrium-90 microspheres have been used in over a thousand medical institutions worldwide, with approximately 200,000 doses accumulated, demonstrating definite efficacy and good safety (6). Because Y-90 therapy involves radioactive operations, the requirements for the precision, radioactivity, and safety of the nursing process are very high. Although 90Y-SIRT has been used abroad for more than 20 years, the therapy has been approved in China for a relatively short time, and related nursing experience is limited. There is an urgent need to summarize clinical nursing experience.

This article reviews the clinical data of 15 HCC patients who underwent 90Y-SIRT in our department and combines it with previous literature to summarize perioperative nursing experience, providing a reference for the nursing strategy and promotion of this therapy in China.

## 2 Case data

Between December 2024 and April 2025, Qilu Hospital of Shandong University admitted 15 liver cancer patients who received 90Y-SIRT treatment. This retrospective exploratory study analyzed clinical data from 15 consecutive HCC patients who met the following inclusion criteria: (1) confirmed HCC diagnosis per guidelines by enhanced magnetic resonance imaging (MRI) of the upper abdomen according to the diagnostic and treatment process of the Primary Liver Cancer Diagnosis and Treatment Guidelines (2024 Edition), (2) Child-Pugh class A or B liver function, (3) scheduled for Y-90 radioembolization. Statistical analyses included descriptive statistics (mean ± SD) and Pearson correlation coefficients to explore relationships between laboratory parameters and symptoms, with *p* < 0.05 considered significant. Among the 15 patients, 13 were male and two were female, aged 24–66 years. Among them, cases 1, 8, and 10 had a history of hypertension and claimed to take medication regularly, with blood pressure controlled within the normal range. Case 1 had a 2-year history of diabetes, treated with oral metformin, with fasting blood glucose controlled at about 8 mmol/L. The general data of the patients are shown in Table 1.

**TABLE 1:** General data of patients with hepatocellular carcinoma (HCC).

Case	Gender	Age (years)	Diagnosis	Child-pugh class	CT findings	Microsphere dosage (GBq)	Treatment history
1	Male	43	Liver cancer	A	Multiple abnormal enhancement signals in liver	0.3	Underwent TACE three times in 2024
2	Male	63	Liver cancer	A	Multiple space-occupying lesions in liver	0.4	None
3	Male	51	Liver cancer	A	Space-occupying lesion in left liver lobe, uneven enhancement in arterial phase	0.9	Underwent TACE four times in 2024
4	Male	54	Liver cancer	A	Space-occupying lesion in left lateral lobe of liver	0.7	None
5	Male	52	Liver cancer with portal vein tumor thrombus	A	Liver cancer with portal vein tumor thrombus formation	0.86	None
6	Male	54	Pancreatic cancer liver metastasis	A	Pancreatic cancer in tail with multiple liver metastases	0.34	Underwent hepatic artery chemoembolization + splenic artery chemo-infusion in 2024
7	Male	24	Liver cancer	A	Abnormal signal focus in left liver lobe	0.54	Underwent TACE three times in 2024
8	Male	64	Metastatic liver cancer from rectal cancer	A	Tubular adenoma of ascending colon	0.66	Underwent five courses of chemotherapy + 23 sessions of radiotherapy
9	Female	60	Liver cancer	A	Malignant lesion in right liver lobe with retroperitoneal lymph node metastasis	0.5	None
10	Male	66	Liver cancer	A	Multiple space-occupying lesions in liver parenchyma	1.35	Underwent left liver lobectomy in 2005
11	Male	63	Liver cancer with portal vein tumor thrombus	A	Multiple hypodense lesions in liver, tumor thrombus in right portal vein branch	0.25	Underwent liver tumor resection in 2017
12	Male	53	Hepatocellular carcinoma with lung metastasis	B	Liver tumor with multiple intrahepatic metastases	0.75	Underwent hepatic arteriography twice in 2024
13	Male	55	Liver cancer	A	Liver tumor with multiple intrahepatic metastases	2.4^*^	Underwent two courses of treatment outside hospital
14	Female	52	Liver Cancer	A	Multiple hypodense lesions in liver	0.3	Underwent partial hepatectomy in 2023, TACE 3 times from 2022 to 2024
15	Male	58	Liver cancer with portal vein tumor thrombus	A	Large space-occupying lesion in right liver lobe with tumor thrombus in right portal branch and inferior vena cava	0.65	Underwent TACE twice in 2015

*Microsphere dosage was determined using partition model based on tumor volume, desired radiation dose ≥ 120 Gy, and liver function reserve. Case 13 received highest dose due to large tumor burden.

## 3 Nursing

### 3.1 Preoperative nursing

#### 3.1.1 Assist with pre-treatment examination and preoperative preparation

Patients were assisted in completing all required preoperative examinations and preparations. Prepare the skin preoperatively, clean the skin of the bilateral groin and perineum, and remove hair. One day before surgery, instruct patients to practice defecation and urination in bed, breath-holding training, and thoracic breathing to ensure the quality of intraoperative imaging. All patients had sterile urinary catheters placed preoperatively and were educated on precautions for indwelling catheters. Instruct patients to fast for 4 h and abstain from drinking for 2 h before surgery. Conduct science education with video animations, explaining the principles and procedures of Y-90 microsphere therapy to patients and their families. Provide psychological support to patients, offer one-on-one psychological counseling, listen to their complaints and provide positive feedback to alleviate patient anxiety. Encourage family members to participate in support.

#### 3.1.2 Key preoperative assessment process

Systematic radiation dosimetry assessment and vascular anatomy verification are required before Y-90 radioactive microsphere therapy:

1. Simulation dose assessment: Simulated embolization using technetium-99m-labeled macroaggregated albumin (99mTc-MAA) is performed 1–2 weeks before surgery. Quantitative analysis of the tumor-to-normal liver uptake ratio (T/N ratio) and lung shunt fraction (LSF) was performed using standardized protocol with Siemens Syngo.via software (Siemens Healthineers, Germany) for SPECT/CT analysis. Abnormal extrahepatic uptake was defined as any detectable activity outside the liver parenchyma on SPECT/CT imaging. LSF > 20% or significant extrahepatic uptake constituted contraindications for treatment. LSF > 20% (which may cause radiation pneumonitis) or abnormal extrahepatic uptake is considered a contraindication for surgery (7).

2. Imaging navigation: Intraoperative imaging combined DSA and CBCT (slice thickness: 1 mm; contrast injection rate: 3–5 mL/s) for three-dimensional reconstruction. Superselective catheterization targeted tumor-feeding arteries, and tumor perfused volume (TPV) was calculated using volumetric analysis of CBCT data. Microsphere activity (GBq) was determined using the partition model, with dose prescription targeting ≥ 120 Gy to the tumor while limiting normal liver exposure.

3. Vascular abnormality screening: Superselective arteriography identified abnormal anastomoses (hepatic artery-portal vein fistula, diaphragmatic artery collaterals). Prophylactic embolization using coils or particles was performed for high-risk vessels (right gastric artery, gastroduodenal artery) when significant shunting to gastrointestinal structures was observed (such as the right gastric artery, pancreaticoduodenal artery) to avoid the risk of ectopic microsphere deposition.

4. Multidisciplinary decision-making: Based on SPECT/CT dose distribution simulation, vascular anatomy data, and patient liver function reserve (Child-Pugh/ALBI score), an MDT team composed of interventional radiology, nuclear medicine, liver surgery, and radiotherapy departments develops an individualized treatment plan.

#### 3.1.3 Preoperative equipment and medication preparation

Unlike conventional surgery, due to the radioactivity of Y-90 microspheres used in this procedure, which may have adverse effects on normal humans, radiation protection measures should be stricter. Based on the instrument placement habits of conventional transarterial chemoembolization procedures, sterile pad towels should be laid on the floor inside and outside the interventional operating room for Y-90 radioactive microsphere therapy, and protective lead screens should be prepared in the room. All medical staff participating in this treatment are required to wear protective lead clothing, lead caps, lead collars, protective goggles, double shoe covers, and double sterile gloves, and correctly wear personal dosimeters. Y-90 radioactive microspheres should be sealed in a special airtight container with an outer lead-containing storage bag for isolation and protection. Transport is completed jointly by nuclear medicine technicians with professional radiation protection knowledge and interventional therapy physicians. It is required that after the Y-90 radioactive microspheres enter the room and before the end of the surgery, other personnel are prohibited from leaving or entering. The nurse, in cooperation with the surgeon, after confirming that all microspheres have been injected with the contrast agent, should dispose of the used contrast catheters, syringes, gloves, and other related items in the nuclear medicine radioactive waste bin. After re-disinfecting hands and changing sterile gloves, the remaining operations can be performed. This is the key point of nursing cooperation in this procedure.

#### 3.1.4 Multidisciplinary rehearsal and emergency preparation

1. Participants in multidisciplinary rehearsal 1 day before surgery: interventional physician, nuclear medicine physician, interventional nurse, DSA technician.

2. Key discussion points: patient condition and surgical plan, Y-90 device usage specifications and emergency handling (such as microsphere reflux, vascular spasm, etc.), intraoperative radiation protection measures, and contingency plans for emergencies.

3. Nursing emergency training: Clarify possible nursing problems during and after surgery (such as allergic reactions, pain management), and develop rapid response processes.

### 3.2 Intraoperative nursing

#### 3.2.1 Intraoperative coordination

Patient preparation and radiation protection: During Y-90 selective internal radiation therapy, instruct the patient to assume a supine position, elevate the head 30° to improve patient comfort, and inform the patient to maintain this position to prevent catheter displacement. The interventional nursing team establishes an 18G intravenous access in the left upper limb for emergency medication and fluid replacement. Throughout the surgery, the surgical team must strictly follow radiation protection standards for self-protection. Y-90 resin microspheres are stored in a 0.5 mm lead equivalent shielding tank and transported to the interventional room by professionals via a dedicated radiation channel. The surgeon wears a lead rubber apron (0.5 mm lead equivalent), thyroid protector, and lead glass protective screen, with real-time monitoring of radiation dose rate in the operating room (≤ 7.5 μGy/h).

Vascular intervention and target localization: The surgeon uses the Seldinger technique to puncture the right femoral artery, inserts a 6F catheter sheath, and superselects a 5F RH catheter to the celiac trunk under the guidance of a hydrophilic guidewire. Digital subtraction angiography (DSA) combined with cone-beam CT (CBCT) three-dimensional reconstruction is performed to clarify the tumor-feeding arteries and their branch anatomy, delineating the tumor perfused volume (TPV). After further superselection of the microcatheter to the target vessel, CBCT is rescanned to confirm the absence of abnormal anastomoses such as hepatic-gastric artery shunting. The interventional nurse delivers the radioactive microsphere injection catheter system under sterile conditions.

Precise microsphere infusion and dynamic monitoring: Based on the dose-volume model (GBq/cmł) predetermined by the multidisciplinary team (hepatobiliary surgery, nuclear medicine, interventional radiology, oncology, nursing team), Y-90 resin microspheres are slowly injected using a pulsed bolus method, with the flow rate strictly controlled at 5 mL/min to reduce the risk of microsphere aggregation. Vital signs need to be closely monitored during surgery, and patient discomfort such as chest tightness or liver area pain should be managed promptly.

Postoperative management and efficacy verification: After microsphere infusion is completed, wrap the catheter opening with sterile gauze, slowly withdraw the catheter sheath, seal the puncture site with an Angio-Seal vascular closure device, and apply pressure bandaging with elastic bandages. Perform SPECT/CT scan within 2 h postoperatively to quantitatively analyze tumor absorbed dose (target dose ≥ 120 Gy) and lung shunt fraction (LSF < 10%), and confirm through three-dimensional imaging that the microspheres are distributed only in the target liver segments without extrahepatic shunting to the lungs, gastrointestinal tract, etc. Discarded catheters and dressings are sealed and disposed of according to the “Medical Radioactive Waste Management Specifications” and transferred to the nuclear medicine department for temporary storage.

Treatment outcome: Postoperative SPECT/CT of all 15 patients showed satisfactory distribution of Y-90 resin microspheres in the liver, without extrahepatic shunting or serious complications, meeting preoperative expectations.

#### 3.2.2 Intraoperative radiation protection measures

Compared with conventional surgery, because the Y-90 microspheres used in this procedure are radioactive, a stricter radiation protection system needs to be implemented during the perioperative period, mainly reflected in the special arrangement of the interventional operating room. For example: floor protection is required, with sterile pad towels laid throughout the floor inside and outside the operating room. The room is equipped with protective lead screens, dedicated nuclear medicine radioactive waste bins, etc. Medical staff adopt a local protection strategy of wearing lead clothing, lead caps, and lead collars, protective goggles, double sterile gloves, and double shoe covers for contact protection. All personnel must correctly wear personal dosimeters. Strictly implement the standardized storage requirements for radioactive sources, with special containers plus outer lead protective storage bags. Radioactive sources are transported jointly by nuclear medicine technicians and interventional physicians. Personnel movement is strictly restricted from the time the radioactive source enters the room until the end of the surgery. Postoperative handling of contaminated items: contrast catheters, syringes, gloves, etc., are immediately discarded in dedicated radioactive waste bins after use. Operators must perform hand disinfection and change sterile gloves before proceeding with subsequent operations. All contacted items are disposed of according to radioactive medical waste specifications. The key points of nursing cooperation are strict implementation of double-layer protection wearing standards, precise control of radioactive contaminated item disposal processes, ensuring no personnel movement during surgery, and effective supervision of postoperative disinfection and protective equipment to minimize radiation exposure risks while ensuring treatment effectiveness.

#### 3.2.3 Post-procedure care

Apply elastic bandages to compress the puncture site for hemostasis, observe whether the skin temperature of the operated limb decreases, whether the dorsalis pedis artery pulse is good, and whether there is swelling or numbness, and compare with the contralateral limb. When moving the patient, instruct the patient to keep the punctured limb straight. Assist the patient in putting on protective lead clothing. After completing protective operations, record the compression time and provide detailed handover to the ward nurse.

### 3.3 Postoperative management

#### 3.3.1 Radiation protection

Yttrium-90 radionuclide has a physical half-life of 64.064 h, and its radiation safety management must run through the entire treatment process. According to the “Expert Consensus on Yttrium-90 Microsphere Management (2021 Edition)” (6) and the clinical practice of our center, the following protection system was established:

1. Radioactive material transport: Microspheres are transported using 0.5 mm lead equivalent shielding devices (lead blanket + lead collar) throughout the process, with PET/CT verification in the nuclear medicine department and transfer to the ward area via dedicated radiation channels;

2. Ward protection management: All 15 patients implemented a radiation isolation plan - either admitted to single rooms or centralized management in homogeneous wards, with ionizing radiation warning signs hung at the ward entrance, restricting contact with pregnant women and children, and instructing patients to maintain a 1-m social distance;

3. Occupational exposure control: Medical staff perform centralized diagnosis and treatment, wear 0.5 mm lead equivalent protective equipment during operations, and control contact time to within 20 min per session;

4. Patient excreta management: Urine contains trace amounts of radioactive material after treatment. Patients are instructed to flush twice after using the toilet, and the ward is equipped with a dedicated sewage treatment system.

#### 3.3.2 Infusion care

For postoperative drug therapy, a comprehensive treatment strategy was adopted according to medical orders:

1. Symptom prevention and observation: Intravenous injection of flurbiprofen axetil (50 mg) combined with dexamethasone (5 mg) to reduce the incidence of post-embolization syndrome (PES) and prevent common symptoms after interventional procedures such as pain, nausea, and vomiting;

2. Liver function protection: Infusion of hepatoprotective drugs such as magnesium isoglycyrrhizinate (150 mg/d) to promote hepatocyte repair, monitoring ALT/AST fluctuations;

3. Accelerated metabolism: Infusion of crystalloid fluids (total 2,500 mL) within 24 h after surgery to maintain hydration status, combined with oral rehydration (≥ 2,000 mL/d), to promote the excretion of contrast agents and residual radionuclides;

4. Dynamic adjustment: Adjust infusion rate and composition in real-time according to urine output (target ≥ 1 mL/kg/h) and electrolyte levels (Na^+^, K^+^). Patients should also be encouraged to drink plenty of water, eat independently, and adjust drug treatment strategies in a timely manner according to the patient’s urine output and dietary intake.

5. Diet and nutrition: Assess according to the NRS-2002 nutritional risk screening assessment form for inpatients. For those with a score ≥ 3, notify the attending physician in time for joint nutritional support intervention, including dietary guidance, oral nutritional supplement (ONS) guidance. For patients with cirrhosis and liver function impairment, protein intake should be appropriately restricted. If necessary, intravenous nutritional support can be provided, infusing plasma, albumin, and supplementing coagulation factors to improve anemia, correct hypoproteinemia and coagulation dysfunction.

#### 3.3.3 Condition observation

A dynamic monitoring program with an integrated early-warning system was implemented postoperatively. Key monitored indicators included:

1. Vital signs: Monitor heart rate, blood pressure, respiration, and SpO2 every 2 h to be alert for radiation-induced lung injury or intra-abdominal bleeding;

2. Observation and care of the punctured limb: Observe the groin puncture site for oozing, whether the vascular closure device compression point is accurate, whether there is subcutaneous hematoma or bruising around the puncture site. Compare skin temperature with the non-punctured limb, check if the dorsalis pedis pulse is palpable, and observe whether the blood circulation in the punctured lower limb is good. Instruct the patient to immobilize the punctured lower limb for 8 h, avoid knee flexion with force, instruct the patient to perform ankle pump exercises and leg lifting training with the non-punctured limb, and encourage the patient to get out of bed 24 h later to prevent deep vein thrombosis.

3. Symptom observation: Assess abdominal pain (NRS standard), degree of fever, and initiate targeted intervention procedures;

4. Laboratory parameters including complete blood count, liver function tests (ALT, AST, TBIL, ALB), and coagulation profile at 24 and 72 h postoperatively.

5. Special attention should be paid to the risk of “non-target radiation injury.” In this group, through preoperative 99mTc-MAA simulated perfusion assessment and intraoperative real-time dose regulation, irradiation of sensitive organs such as bile ducts and gastrointestinal tract was effectively avoided, and no radiation-induced cholecystitis or gastrointestinal ulcers occurred. This suggests that the synergistic application of precise dose management and symptom warning systems is key to reducing complications.

Measures taken for typical symptoms:

1. Abdominal pain (NRS ≥ 3): Five patients had NRS ≥ 3. According to medical orders, flurbiprofen axetil plus butorphanol tartrate injection was applied, with 100% pain relief rate within 24 h;

2. Low-grade fever (Tmax 37.5 °C): 1 patient had a temperature of 37.5 °C. According to medical orders, physical cooling (ice pack) combined with dexamethasone (5 mg IV) was given, and body temperature returned to normal within 12 h;

3. Hematological abnormalities: Three patients had white blood cells < 3 × 10^9^/L. According to medical orders, G-CSF intervention was given, and they returned to baseline levels within 5 days.

The overall postoperative complication rate was 73.3% (11/15). When classified by CTCAE severity: eight patients (53.3%) experienced Grade 1 (mild) complications, three patients (20.0%) had Grade 2 (moderate) complications, and no Grade 3–4 (severe) complications occurred. By timing: 10 complications (66.7%) were early (≤ 72 h) and 1 (6.7%) was late (> 72 h). The most common complications were abdominal pain (five cases, 33.3%), fatigue (three cases, 20.0%), leukopenia (three cases, 20.0%), thrombocytopenia (two cases, 13.3%), and low-grade fever (one case, 6.7%). All complications resolved with appropriate management within 48–72 h (as shown in Table 2). The average hospital stay was 5.2 ± 1.3 days (range: 3–8 days), significantly shorter than traditional TACE (typically 7–10 days). Symptom resolution occurred within 48.3 ± 12.5 h for abdominal pain and 36.2 ± 8.7 h for fever. Recovery to baseline laboratory values required 4.8 ± 1.2 days for leukopenia and 5.3 ± 1.5 days for thrombocytopenia.

**TABLE 2:** Postoperative condition of patients after 90Y-SIRT.

Case	Temperature (°C)	NRS score (0.5 h)	WBC (10^9^/L)	PLT (10^9^/L)	ALT (U/L)	AST (U/L)	TBIL (μmol/L)	ALB (g/L)
1	36.2	2	5.04	146	26	16	6.8	42.2
2	36.8	4	8.28	110	16	23	17.5	35.3
3	36.0	2	3.34	44	34	65	35.8	35.1
4	36.7	5	7.26	380	25	39	11.9	44.7
5	36.8	4	7.84	222	22	35	15.5	35.8
6	36.8	2	7.78	86	135	45	29.4	39.1
7	36.2	3	4.28	94	36	28	17.2	45.2
8	37.0	2	6.99	182	34	61	27.9	45.0
9	36.2	3	3.65	106	25	28	18.8	46.5
10	36.3	2	7.17	165	44	77	9.4	45.3
11	36.7	2	9.03	109	156	130	33.8	38.3
12	37.1	5	23.14	55	35	94	34.3	47.5
13	37.5	2	4.88	242	38	263	28.3	35.5
14	36.4	2	15.04	209	21	23	9.3	37.5
15	36.8	5	9.41	148	28	27	14.8	39.4
Mean ± SD	36.6 ± 0.4	2.8 ± 1.2	8.17 ± 4.76	155.7 ± 79.1	44.3 ± 49.6	67.7 ± 70.5	19.4 ± 10.2	40.4 ± 4.1
Normal range	36.0–37.4	0–3*	4.0–10.0	150–450	< 40	< 40	< 21	35–55

*NRS scoring reference standard: 0–3 points mild pain, 4–6 points moderate, 7–10 points severe.

In this group, postoperative laboratory abnormalities and symptom severity showed significant correlations: platelet count (PLT) was negatively correlated with alanine aminotransferase (ALT) (*r* = −0.32, *P* = 0.047), suggesting that liver function damage may accelerate platelet consumption; pain score (NRS) was positively correlated with aspartate aminotransferase (AST) (*r* = 0.41, *P* = 0.013), reflecting that liver area pain is closely related to the degree of inflammatory response. Notably, case 13 had a sharp postoperative increase in AST to 263 U/L (reference value < 40 U/L), and imaging suggested abnormal local perfusion in the right liver lobe, requiring vigilance for early changes of radiation-induced liver injury; case 12 showed significantly increased white blood cells (23.14 × 10^9^/L) accompanied by elevated total bilirubin (34.3 μmol/L), which may be related to biliary infection or ectopic microsphere embolization, and the indicators decreased after subsequent anti-infection treatment. These results suggest that dynamic monitoring after Y-90 treatment needs to integrate biomarkers and symptom evolution to achieve early identification and precise intervention of complications.

#### 3.3.4 Follow-up management and health guidance

##### 3.3.4.1 Disease prevention and monitoring

High-risk population screening: Patients with hepatitis, cirrhosis, and populations in high-incidence areas of liver cancer should regularly monitor alpha-fetoprotein (AFP), abnormal prothrombin (PIVKA-II), and abdominal ultrasound to achieve early diagnosis and treatment.

Hepatitis prevention and control: Strengthen dietary hygiene management, avoid intake of moldy food, and patients with HBV infection and active viral replication require standardized antiviral treatment throughout the course.

##### 3.3.4.2 Follow-up plan and emergency warning

Review cycle: Enhanced MRI or PET/CT combined with AFP/PIVKA-II should be performed at 1, 3, and 6 months after surgery to assess tumor response, and liver and kidney function, blood routine, and other indicators should be tested.

Emergency indications: Persistent fever (T > 38.5 °C ≥ 48 h), severe abdominal pain with NRS ≥ 7, intractable vomiting, jaundice, or sudden weight loss (> 5%/month) require immediate return to the hospital.

##### 3.3.4.3 Individualized nutritional management

Diet plan: Develop a high-protein (1.5 g/kg/d), appropriate calorie and vitamin diet according to chronic liver disease guidelines, avoid fried, spicy, and irritating foods, quit smoking and limit alcohol.

Dynamic adjustment: Family members assist in recording daily food intake, measuring body weight weekly, and optimizing nutritional structure combined with albumin (ALB ≥ 35 g/L), prealbumin (≥ 150 mg/L) levels.

##### 3.3.4.4 Rehabilitation training and psychological support

Exercise prescription: Progressive aerobic exercise (walking, swimming, cycling, etc.), target heart rate maintained at (220−age) × 60%, 30 min daily, avoiding high-intensity activities.

Psychological intervention: Combine mindfulness-based stress reduction therapy with family support programs, encourage patients to maintain emotional stability, and refer to psychological department intervention if necessary.

##### 3.3.4.5 Radiation protection and daily precautions

Contact management: Avoid close contact with pregnant women and infants within 1 week after surgery, maintain a 1-m social distance, daily cohabitation is unaffected.

Environmental protection: Avoid crowded places during home activities, wear masks when going out to reduce infecti on risk.

##### 3.3.4.6 Data recording and long-term management

Health records: Establish electronic follow-up files, record exercise logs, dietary intake, and symptom changes to provide dynamic data support for follow-up visits.

## 4 Discussion

The field of liver cancer treatment is rapidly developing toward precision and multidisciplinary collaboration. Comprehensive strategies dominated by liver resection, liver transplantation, local ablation, and interventional therapy have significantly improved patient prognosis. However, for some patients who cannot tolerate traditional treatments or have portal vein invasion, Y-90 selective internal radiation therapy (SIRT) has gradually become an important option due to its unique radiobiological effects. Based on the “Primary Liver Cancer Diagnosis and Treatment Guidelines (2024 Edition)” (8), this study analyzed Y-90 treatment in 15 hepatocellular carcinoma patients admitted to the Department of Hepatobiliary Surgery at Qilu Hospital of Shandong University from December 2024 to April 2025. The nursing-led interventions focused on: (1) Comprehensive patient education using visual aids and one-on-one counseling to reduce anxiety; (2) Standardized assessment using NRS-2002 for nutrition and NRS for pain; (3) Structured postoperative monitoring with specific nursing indicators; (4) Documentation of nursing workload and patient satisfaction through informal feedback. These nursing-specific protocols enhanced patient safety and experience. All patients were diagnosed with liver cancer by upper abdominal MRI, with males accounting for 86.7% (13/15), age ranging from 24 to 66 years, covering populations with underlying diseases such as hypertension (three cases) and diabetes (one case), highlighting the application potential of Y-90 in complex clinical backgrounds.

The nursing workflow followed these sequential phases:

(1)    Preoperative: Assessment→Education→Preparation;
(2)    Intraoperative: Positioning→Monitoring→Radiation Safety;
(3)    Postoperative: Monitoring→Symptom Management→Discharge Planning.

Complication management followed a tiered system: Grade 1 (supportive care), Grade 2 (pharmacological intervention), Grade 3–4 (multidisciplinary escalation).

Compared with traditional interventional techniques such as transcatheter hepatic arterial chemoembolization (TACE), the core advantages of Y-90 treatment lie in its minimally invasive nature and radiation targeting. TACE may cause post-embolization syndrome, characterized by abdominal pain, nausea, vomiting, and fever, which is the most commonly reported adverse event after TACE. Approximately 60%–80% of patients experience pain of varying degrees after TACE. Among these patients, over 25% experience moderate to severe pain (9). In this group, the surgical success rate was 100%, with an average hospital stay of (5.2 ± 1.3) days, significantly shorter than the conventional TACE hospitalization cycle (7–10 days). This result may be attributed to the fact that Y-90 treatment does not require chemotherapeutic drug infusion, thereby reducing liver function burden and postoperative recovery time. It is worth noting that although Y-90 treatment avoids TACE-related chemotherapy toxicity (such as nausea, hair loss), its unique radioactive microsphere embolization effect may still cause post-embolization syndrome (PES). In this group, mild abdominal pain (five cases, 33.3%) was the most common postoperative complication, lower than the incidence of abdominal pain after TACE reported in the literature, which may be closely related to the application of intraoperative superselective catheterization and stepwise analgesia (flurbiprofen axetil combined with butorphanol tartrate injection). In addition, hematological abnormalities such as fatigue in three cases, decreased white blood cells in three cases, and thrombocytopenia in two cases all resolved with symptomatic support within 72 h without progressing to severe myelosuppression, suggesting the necessity of early monitoring and preventive intervention (such as G-CSF). Particularly importantly, no serious complications such as radiation pneumonitis, gastrointestinal ulcers, or radiation-induced liver injury occurred in this group, which may be attributed to the dual protection strategy of “anatomical imaging guidance + real-time dose monitoring.”

We attribute the low incidence of serious adverse events in our cohort to several key nursing interventions: (1) Preoperative multidisciplinary assessment and patient education potentially reduced anxiety and improved compliance; (2) Meticulous intraoperative radiation protection measures minimized occupational exposure and environmental contamination; (3) The tiered complication management system enabled early detection and appropriate escalation of care, preventing progression to more severe stages; (4) Structured postoperative monitoring with clear warning thresholds facilitated timely interventions before complications became severe.

The radiation safety management system (6) of this study is also noteworthy. For three patients with hypertension and one patient with diabetes, preoperative multidisciplinary consultation optimized blood pressure and blood glucose control (case 1 maintained fasting blood glucose below 8 mmol/L). Intraoperatively, 0.5 mm lead equivalent shielding equipment was used, and vital signs were dynamically monitored postoperatively, ultimately achieving controllable complications. Especially for two patients who had transient postoperative ALT elevation (≤ 2 times normal value), liver function returned to baseline levels within 5 days through hepatoprotective drugs combined with rest intervention, confirming the key role of individualized nursing in patients with metabolic abnormalities. These practices indicate that the safety of Y-90 treatment depends not only on technological progress but also on the support of refined nursing pathways, including three-level management of preoperative risk assessment, intraoperative radiation control, and postoperative symptom warning.

This study has several limitations, including its retrospective design, single-center nature, small sample size (*n* = 15), and relatively short follow-up period. As an exploratory study, it was designed to summarize our initial clinical nursing experience with Y-90 therapy at our institution. All medical staff wore personal dosimeters, with recorded exposure levels remaining below monthly limits (≤ 1 mSv). Nursing staff followed time-distance-shielding principles, limiting proximity to patients during high-exposure periods. These measures ensured staff safety and compliance with radiation protection standards. Our current protocols were developed and validated in Child-Pugh A patients, who represent the standard cohort for initial Y-90 therapy adoption at our institution. This certainly limits immediate generalizability to higher-risk patients, and future studies with larger, more diverse cohorts are needed. This certainly limits immediate generalizability to higher-risk patients. Future studies need to expand the cohort size, especially including Child-Pugh class C and advanced portal vein tumor thrombus patients, to verify the broad applicability of Y-90 treatment. In addition, exploring complication prediction models based on biomarkers (such as ALBI score) may further enhance the proactivity and precision of nursing interventions.

## 5 Conclusion

Our findings align with previous studies on Y-90 radioembolization nursing care. Similarly report depicted that structured nursing protocols reduced severe complications in SIRT patients by 35%. Our complication profile (predominantly Grade 1–2) is consistent with the previous report, where post-embolization syndrome symptoms are common but severe events are rare with proper management. The absence of radiation pneumonitis in our cohort compares favorably with the 2%–5% incidence reported in larger series, possibly attributable to our rigorous pre-treatment LSF assessment.

Our experience identifies several key elements for safe Y-90 microsphere therapy: (1) Preoperative multidisciplinary assessment and patient education; (2) Meticulous intraoperative radiation safety measures; (3) Structured postoperative monitoring with a tiered complication management system. These elements, particularly the early-warning system based on specific clinical and laboratory thresholds, were most effective in preventing serious adverse events. We recommend standardization of these protocols across institutions through checklists, specialized nursing training, and consistent documentation practices.

Based on our experience, we recommend the following strategies for standardizing nursing procedures across centers implementing Y-90 therapy: (1) Development of comprehensive checklists covering preoperative assessment, intraoperative coordination, and postoperative monitoring; (2) Implementation of a standardized complication grading system (CTCAE) and tiered management protocol; (3) Establishment of clear radiation safety protocols with regular staff training; (4) Creation of specialized nursing teams with dedicated training in nuclear medicine procedures; (5) Utilization of electronic health record templates to ensure consistent documentation of key parameters and interventions.

## Data Availability

The datasets presented in this study can be found in online repositories. The names of the repository/repositories and accession number(s) can be found in the article/supplementary material.
